# Deep Learning-Based Extraction of Biomarkers for the Prediction of the Functional Outcome of Ischemic Stroke Patients

**DOI:** 10.3390/diagnostics13243604

**Published:** 2023-12-05

**Authors:** Gonçalo Oliveira, Ana Catarina Fonseca, José Ferro, Arlindo L. Oliveira

**Affiliations:** 1 NeuralShift, 1000-138 Lisbon, Portugal; 2 INESC-ID, Instituto Superior Técnico, 1000-029 Lisbon, Portugal; 3 Faculdade de Medicina, Universidade de Lisboa, 1649-028 Lisbon, Portugal; acfonseca@medicina.ulisboa.pt (A.C.F.); jmferro@medicina.ulisboa.pt (J.F.)

**Keywords:** deep learning, ischemic stroke, logistic regression, convolutional neural networks, functional outcome, computed tomography

## Abstract

Accurately predicting functional outcomes in stroke patients remains challenging yet clinically relevant. While brain CTs provide prognostic information, their practical value for outcome prediction is unclear. We analyzed a multi-center cohort of 743 ischemic stroke patients (<72 h onset), including their admission brain NCCT and CTA scans as well as their clinical data. Our goal was to predict the patients’ future functional outcome, measured by the 3-month post-stroke modified Rankin Scale (mRS), dichotomized into good (mRS ≤ 2) and poor (mRS > 2). To this end, we developed deep learning models to predict the outcome from CT data only, and models that incorporate other patient variables. Three deep learning architectures were tested in the image-only prediction, achieving 0.779 ± 0.005 AUC. In addition, we created a model fusing imaging and tabular data by feeding the output of a deep learning model trained to detect occlusions on CT angiograms into our prediction framework, which achieved an AUC of 0.806 ± 0.082. These findings highlight how further refinement of prognostic models incorporating both image biomarkers and clinical data could enable more accurate outcome prediction for ischemic stroke patients.

## 1. Introduction

According to the World Stroke Organization, each year, there are over 7.6 million new ischemic strokes, which corresponds to more than 62% of all strokes. Of these annual ischemic strokes, 3.3 million result in death. Additionally, ischemic stroke patients collectively lose more than 63 million healthy years, due to stroke related death and disabilities, each year [1].

Naturally, both the patient and family want to have early information regarding stroke prognosis. Accurate early prediction of post-stroke disability is crucial to both the patient and family to inform actions that should be taken to adapt to a new reality. Additionally, the administration of the thrombolytic drug usually given to these patients can only be done in a limited time frame, and is not risk free [2]. Therefore, in a future where a post-stroke functional outcome predictor is available, these risk factors could be better considered by physicians, which might also allow the use of personalized treatments [3].

**Figure 1:**
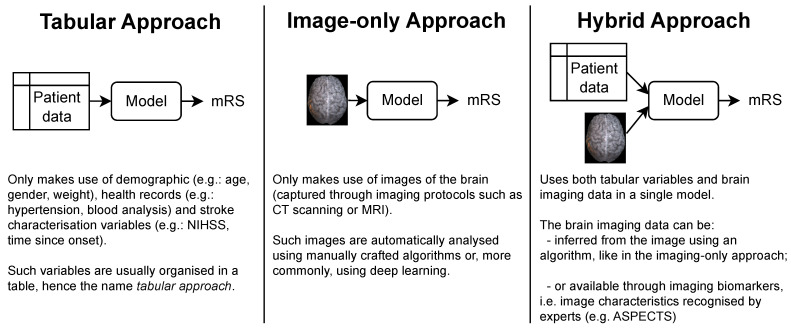
The three main approaches for predicting the mRS. Notably, each approach is characterized only by the type of data it uses. In particular, no assumption is made on how these data are given to the model or how it is processed. This means that the brain imaging data can be the raw brain scans or a series of variables that describe it (for example, image biomarkers).

The patient’s functional outcome is commonly considered three months after onset and measured by the modified Rankin Scale (mRS), which is an integer scale that goes from zero to six, where zero corresponds to full independence and six corresponds to death [4,5]. Several models have been proposed by the medical and machine learning (ML) communities to predict this variable. In this work, we categorized these studies into tabular, image-only and hybrid approaches, following the organization proposed by Oliveira et al. [5]. As shown in Figure 1, each approach gets its name from the type of data ingested:

1. **Tabular approach:** only demographic, health records and stroke characterization variables;
2. **Image-only approach:** only brain imaging data;
3. **Hybrid approach:** both tabular variables and brain imaging data.

Even though the proposed definitions of the image-only and hybrid approaches are compatible with any kind of brain imaging protocol, this study was only concerned with data obtained from computed tomography (CT) scans. In particular, we focused on non-contrast CT scans (NCCT), the recommended initial scan procedure for stroke investigation [6], and its contrast enhanced variant, CT angiography (CTA), which enables the visualization of the brain arteries.

In terms of the complexity of the models that have been proposed for each approach, the tabular models are by far the simplest. Since their only input is a series of patient variables, they do not require an analysis of the brain scans, which either requires the attention of human experts or the use of sophisticated imaging algorithms, usually involving deep learning.

While simple, the tabular models do not have access to imaging data available in brain scans, such as head CTs. Such scans are collected as part of standard patient care [6], and are known to have relevant information for the prediction of patients’ functional outcome, despite the fact that early admission brain CT scans of ischemic stroke patients only exhibit subtle visual changes [7]. For example, lower ASPECT scores (a score used to systematize the evaluation of the brain damage seen in NCCTs [8]) are correlated with poorer outcomes. Also, the presence and location of vessel occlusions, as well as infarct size, can be estimated from CTA scans and both these variables are correlated with the mRS [7,9]. These findings support the use of the image-only and hybrid approaches (if the brain image scans did not contain predictive information, there would be no point in incorporating them in the models). However, image-only models often underperform relative to the other approaches, and the hybrid models are usually only marginally better than their tabular counterparts.

To better understand the underperformance of the image-only approach and the apparent weak contribution of the imaging data in the hybrid approach, we conducted a series of experiments that test several different deep learning models in these two approaches. In the image-only approach, we tried architectures never before tried in this particular task, like the Siamese network (SN) and multiple instance learning. In the hybrid approach, we show how a SN can be used to predict the presence of occlusions in CTA scans which, in turn, can improve the mRS classification performance. We call this occlusion variable an imaging biomarker, i.e., a characteristic that can be objectively measured and evaluated from medical images (another example of such biomarkers is the ASPECT score [8]).

Our results support the results previously reported by other studies, i.e., hybrid models are only marginally better than tabular models, and both are better than image-only models. We conclude by presenting a discussion on the challenges of using brain imaging data for the mRS prediction task and how future models that make use of these data can be improved. In particular, our hypothesis is that using CT scans to first extract image biomarkers as an intermediate step to the mRS prediction leads to a better defined optimization problem and also results in models that are more interpretable.

## 2. Literature Review

The literature review is divided into two subsections. Section 2.1 describes studies that have been previously done regarding the prediction of mRS. The selection of studies here presented includes examples of previously proposed tabular, image-only and hybrid models. Section 2.2 presents other studies related to deep learning applied to the analysis of CT scans (not necessarily applied to the mRS prediction task). We mention these studies because they motivated some of the experiments we performed.

### 2.1. Modified Rankin Scale Prediction

Clinical models are simple algorithms, developed by the medical community, that predict the functional outcome usually from no more than ten variables using simple arithmetic, as they are meant to be computed by humans [3]. The ASTRAL [10], DRAGON [11] and THRIVE [12] scores are examples of such models (following the terminology set in the previous section, ASTRAL [10] is a tabular model, while DRAGON [11] and THRIVE [12] are hybrid models, as they have imaging biomarkers as input).

Machine learning (ML) models are algorithms that can learn from and make predictions on data. They do this by learning the relationships, patterns, and insights contained within the data during the learning (also known as “training”) process. After training, the resulting model can then be used to make prediction on new (unseen) data, a process usually known as “inference”. Because both the training and inference stages are meant to be done by a computer, the ML models often include larger subsets of input variables and also combine them in more complex ways than the clinical models. The term “machine learning” is a broad expression that encompasses both deep learning, which is focused on deep neural networks, as well as “classical” ML models, which are often simpler but also more interpretable (it is easier to understand the resulting trained model). Monteiro et al. [3] trained several classical machine learning models like logistic regressions and random forests on various subsets of tabular variables, and found them to be statistically significantly better than the DRAGON [11] and THRIVE [12] models.

One example of an image-only study is Hilbert et al.’s work [13], where the mRS was predicted from CTA scans. Like all CT scans, these are 3D images of the brain, but the authors transformed these volumes into 2D images by using maximum intensity projection (MIP) [14]. They then used a ResNet [15], adapted with receptive field neural networks (RFNNs) [16], to avoid overfitting, as their prediction model. The MIP technique highlights brain arteries in the axial plane which facilitates the detection of occlusions. Indeed, by analyzing the activation mappings of their network, the authors noticed they tended to focus on these occluded arteries.

Unlike Hilbert’s work [13], which used 2D images as input, most studies process the whole CT scans using 3D convolutional neural networks (CNN). This idea was pioneered by Bacchi et al. [17] with the use of a custom 3D CNN, composed of eight layers, that gathered features from NCCTs scans that were later concatenated with features from clinical and demographic variables to make a prediction. Samak et al. [18] also developed a custom CNN to encode NCCT scans and introduced the use of data augmentations, a more thorough pre-processing, focal loss [19] and attention mechanisms [20]. In another work, Samak et al. developed the feature matching auto-encoder (FeMA) [21], another custom CNN that not only predicted the mRS from admission NCCT scans, but also estimated how these scans would look one week later.

In Brugnara et al. [22] and Ramos et al. [23], statistical tests were employed to compare tabular models with hybrid models. In Brugnara’s work, they tested if adding the acute ischemic volumes and ASPECTS biomarkers would improve an otherwise tabular model. They note that despite both variables being strong independent predictors of the target 90 day mRS, there was no clear advantage in adding either of them (nor both) to the model. In Ramos’s work, they tried adding imaging data by using radiomics features and by using deep learning features from a 3D ResNet 10 encoder [15]. In either case, they concluded that adding imaging features did not improve their models’ performance.

For a more in-depth analysis of these studies, as well as a more thorough review of the various previously published image-only and hybrid models studies, please refer to Oliveira et al. [5].

### 2.2. Deep Learning for Brain Imaging

First, in Barman et al.’s work [24], the authors developed a custom CNN they named DeepSymNet to detect strokes in CTA scans. DeepSymNet is a Siamese network [25], which is a type of neural network with two encoders that accept two different inputs whose representations are then compared to produce a prediction. This structure can be seen as an inductive bias useful for comparing inputs that are very similar. In this context, this convenient prior is used to compare the brain hemispheres of the same CTA scan, whose symmetry can become compromised when a stroke occurs. The authors obtain this by splitting the brain across the mid midsagittal plane and then computing representations of each hemisphere with an encoder composed of four stacked 3D inception modules (IM) [26] and their feature maps compared using the L1 difference. Like in Hilbert et al.’s work [13], the authors also analyzed their network activation maps, and noticed that the model was relaying on the detection of occlusions to make its prediction.

Second, Ilse et al. [27] proposed the idea of “deep multiple instance learning”. In a traditional machine learning setting, a model has an instance as input and predicts its label. In the multiple instance learning (MIL) setting, the model has a bag of instances as input and predicts the label of the bag. Each individual instance has its own label, but this label is not available for training. A bag is given the positive label if it contains at least one positive instance, and is given the negative label otherwise [28]. Ilse et al. [27] proposed to decompose the MIL problem into three steps, each parameterized with a neural network, building a flexible end-to-end trained model. The first step is responsible for creating a representation of each instance (when dealing with images, this is usually a CNN encoder). The second step is responsible for aggregating all the instance representations into a single representation of the whole bag. And the third and final step outputs a prediction from this aggregated representation. The aggregation function is usually just the element wise mean or max of the instance representations, but the authors also proposed an attention based aggregation function that allows the model to dynamically choose which instances it should pay more attention to. The authors applied this idea to a histology classification problem, where each instance is a patch of the original image and showed how the attention model ended up focusing on malignant patches, providing a sort of *soft segmentation*, even though no segmentation ground truth labels were given during training.

The MIL paradigm has also already been applied to CT scans. Remedios et al. [29] developed a network to perform hemorrhagic stroke detection, where the axial slices of NCCT scans were modeled as instances, with the goal of detecting which axial slices contained hemorrhage signs (if any). They gathered instance representations using a ResNet 34 encoder [15] and aggregated them using the max pooling aggregator (element wise max). This work also analyzed the data requirements to enable model generalization and concluded that at least 400 training examples were needed [29].

## 3. Materials and Methods

### 3.1. Dataset

This study analyzed the patients collected in the context of the PRECISE study (“PRECISEMED”, https://www.precisemed.org/ accessed on 23 August 2022). This was a research study that included adult ischemic stroke patients from 2016 to 2019, and whose goal was to advance the Portuguese health care systems towards the implementation of precision medicine practices. The inclusion criteria for this study was patients with ischemic stroke less than 72 h from onset and age greater than or equal to 18 years old. The exclusion criteria were active neoplasm, previous cerebral revascularization surgery, and a Rankin score greater than or equal to five. Consecutive patients were recruited from Hospital de Santa Maria (80% of the patients in the dataset), Hospital Egas Moniz, Hospital Pulido Valente and Hospital de São José. Participation in the study was voluntary and meant no changes in the way patients were treated, apart from an increase in blood collection (from 15 to 50 milliliters).

We collected data regarding demographic parameters, previous medication, time of stroke onset and stroke characteristics, vascular risk factors, medical history, blood results at admission including glucose and international normalized ratio (INR), brain imaging at admission (brain CT and CTAs), acute treatments performed, etiological investigation exams performed, secondary prevention treatments, and functional outcome evaluated using the Rankin score at discharge, three months and one year after stroke onset.

For the purpose of the current study, we analyzed the subset of patients that were admitted within the first 24 h after the stroke onset. This study was approved by the ethical committee of the participating hospitals. All patients or legal representatives signed an informed consent. The tabular data used in these analyses was downloaded from the PRECISE database on the 29 May 2022, and a summary of its characteristics can be seen in Table 1. This selection resulted in a dataset of 743 individuals. However, different subsets of it were used for each experiment, depending on the data available. For example, for the image-only models, only the subset of patients that had a baseline NCCT exam were used. We provide additional details about the subset of patients used in the image-only approach, hybrid approach and occlusion prediction experiments in Section 3.3.1, Section 3.3.2 and Section 3.3.2.1, respectively.

### 3.2. CT Preprocessing

Only a small portion of the CT scans is useful, as the skull and soft tissues like muscle and fat are not relevant in the context of ischemic stroke prognosis. Also, patients often have their head in slightly different orientations, which adds unwanted heterogeneity. The removal of the skull and other irrelevant tissues can be done with skull stripping (a process also known as “brain extraction”). The head position correction can be done with template registration, i.e., the matching of the scan with a common template, usually the MNI152 [30]. Some authors perform both skull stripping and template registration [13,21,24], others perform only skull stripping [23,29] and others perform no preprocessing at all [17,18]. As this normalization process is not standardized, a custom pipeline was developed and made publicly available (https://github.com/GravO8/CT-preprocess, accessed on 12 October 2023). First, scans were converted from DICOM to NIfTI, using dcm2niix [31]. Then, using FLIRT [32,33] from the FSL, NCCT and CTA scans were registered to the MNI152 T1 [30] 2 mm and 1 mm templates, respectively (chosen according to the slice thickness of the respective modality). Besides correcting the head orientation of the scans, this step also removes most of the air pixels on the side of the head, by resizing the NCCTs to a 91 × 109 × 91 volume (and CTAs to double of that). A validated algorithm, adapted from the FSL’s BET [34,35,36], was used to extract only the brain from the NCCT scans. For CTA scans, a custom algorithm based on thresholding and morphological operations was used instead. After being preprocessed, scans were visually inspected and discarded if any preprocessing issues were found. Not all patients in this study have a CT scan in the repository and so, of the NCCTs available, 465 were kept and 103 were dropped and of the CTAs available, 361 were kept and 6 were dropped.

Finally, the size of the NCCT training set was increased fourfold by using data augmentations. MSP mirror, random rotations (of at most 10°), random elastic deformation (with seven control points, max displacement of 7.5 and linear interpolation) and random Gaussian noise addition (with mean 5 and standard deviation 2) were performed offline, using the TorchIO library [37]. Some examples of the application of these augmentations are available in Figure A2.

### 3.3. mRS Prediction Models

In this work, we tested three different architectures for the image-only approach. Additionally, we tried various hybrid models based on logistic regressions and compared them to the ASTRAL [10] clinical classifier. This section first explains our image-only experiments which use NCCTs as input, then our hybrid experiments. which used CTAs and other patient variables as input. All these (image-only and hybrid) experiments were modeled as a binary classification problem by splitting the mRS target variable, 3 months after stroke into good outcome (mRS ≤ 2) and poor outcome (mRS > 2) classes, as is usually done in the literature [5].

#### 3.3.1. Image-Only Approach

**Figure 2:**
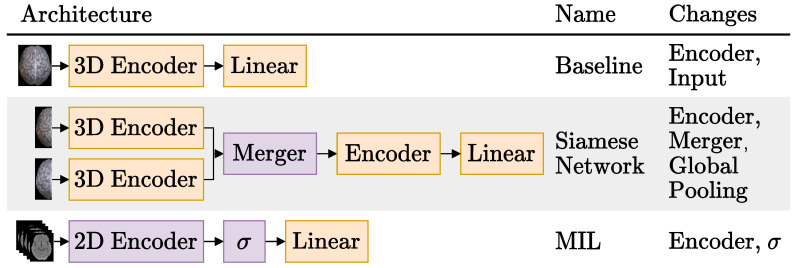
Schematic representation of the three brain imaging only architectures tried. The local changes tried for each architecture are listed on the last column. For more details on the different image-only Siamese models experimented, please refer to Section 3.3.1.2. The σ represents the MIL aggregation step, following the notation used by Ilse et al. [27]. Blocks in orange are updated during training, and blocks in purple may or may not be updated during training (depending on the experiment).

In Figure 2, we show the three architectures that we experimented with for the image-only approach. These experiments were done with the 465 NCCTs scans available: 365 in the training set, 40 in the validation set and 60 in the test set, with a stratified split. Models were trained three times (with different random weight initializations, unless otherwise stated) for 300 epochs, using the Adam optimizer [38], with binary cross entropy, a batch size of 32, weight decay of 0.0001 and learning rate of 0.0005. The best validation set $F_{1}$-score was used to selected the weights of the model loaded to evaluate the test set. The final layer of all three architectures tried is a linear layer with one output neuron, activated with a sigmoid. We now explain the details specific to each individual architecture family: Baseline, Siamese Network and MIL, in this order.

##### 3.3.1.1. Baseline

The first row shows the Baseline model, which uses a 3D CNN to generate features from the whole CTs volume and is the most commonly used architecture in the literature [17,21,23,24,29] to process CT scans. As the rightmost column suggests, the Baseline experiments consisted in trying different encoders and model inputs.

The five encoders tried were a 3D version of the ResNets [15] 18, 34 and 50, the hemisphere encoder proposed in the DeepSymNet [24] and a custom 3D CNN. The 3D ResNets were created from their traditional 2D counterparts by replacing the 2D convolution, batch normalization and pooling operators with their 3D equivalents. The DeepSymNet encoder was designed for 29 × 73 × 20 scans, and has four stacked 3D IMs [26] with 64 filters of each size. These many filters proved to be too much for the larger 91 × 109 × 91 NCCTs available in the present study. Thus, to keep the computational complexity of the network manageable, four filters were used on the first IM and 16 on the next three. Additionally, as the IMs preserve the spatial resolution of their input, an average pooling layer of stride two was added after the first IM to reduce the spatial dimension of the feature maps.

On the other hand, the ResNets drastically reduce the spatial dimension of the feature maps, like other networks trained on ImageNet do [15,39,40]. In practice, 3D ResNets reduce the original 91 × 109 × 91 NCCTs into a 3 × 4 × 3 volume, by the end of the encoder. This and the fact that updating the ResNets into 3D models significantly increases their parameter count motivated the inclusion of a custom 3D CNN as well. This custom CNN, with just 74 K parameters, has only four convolutions with 8, 16, 32 and 64 filters, in this order, all followed by a batch normalization layer and a ReLU activation. The first and third convolutions have a stride of one, and the other two have a stride of two.

**Figure 3:**
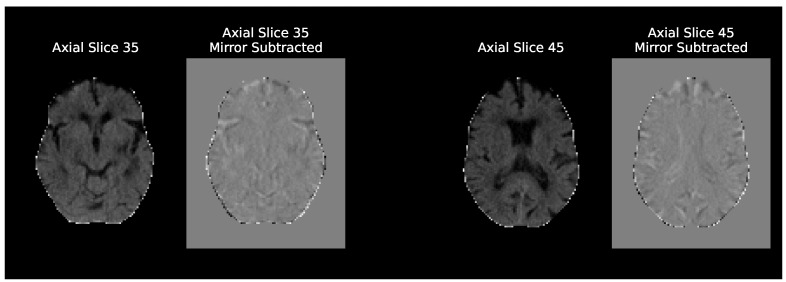
Comparison of axial slices of an NCCT scan given as input in the baseline and baseline mirror experiments. The axial plane is only used to better visualize the anatomical differences of the two approaches, as all the baseline architectures were trained with the whole 3D volumes. These are MNI152 registered scans and the axial slice numbers shown are only valid for the 2 mm template.

Regarding the different model inputs, two different inputs were tested: the preproposed NCCT scans and these scans subtracted by their mirrored (along the MSP) version. These experiments are named simply “Baseline” and “Baseline Mirror” respectively. The idea behind Baseline Mirror is to impose the brain symmetry inductive bias the Siamese networks enjoy in their architecture, on the data itself. Simply subtracting the mirrored scans highlights any differences in the brain hemispheres, either relevant like the ischemic tissue changes or irrelevant, like those caused by imperfect MSP symmetry due to imperfect registrations. Figure 3 compares two axial slices of a NCCT scan in the Baseline and the Baseline Mirror approach.

##### 3.3.1.2. Siamese Network

Like the Baseline experiments, the Siamese experiments had the whole NCCT volumes as input. However, in this case, the volumes were cut in half along the MSP axis and each hemisphere was encoded separately.

**Figure 4:**
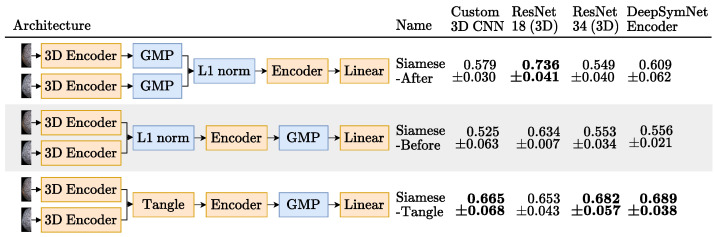
The Siamese networks architectures tried in the image-only approach and their respective results test set mean ± standard deviation AUC, over the 3 runs, when using GMP. For each encoder, the highest average AUC is highlighted in bold. Blocks in orange are updated during training and blocks in blue are not trainable.

**Figure A1:**
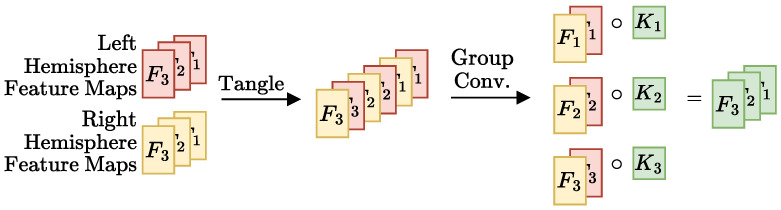
Schematic representation of the tangle operation proposed. Like the L1 norm in the Siamese-Before approach, this function has the two hemisphere feature maps as input and outputs a set of feature maps with the same dimensions of the input maps. The first step is the tangling itself that joins the input maps into a single output tensor such that *i*th left and right hemisphere maps go to the 2ith and (2i+1)th positions in the output tensor, respectively. Group convolutions are a generalization of convolutions where the kernels are divided into equal sized groups (“normal” convolutions are just group convolutions with a single group). The AlexNet [57] used convolutions with 2 groups, with half of the kernels in one GPU and the other half on another, due to limited memory. Here, group convolutions are not used because of memory constraints, but rather to make it so that each *i*th maps pair have their own kernel, not shared by the other pairs. This is done by specifying the number of groups to be the number of feature maps (3, in the schematic example).

**Figure A2:**
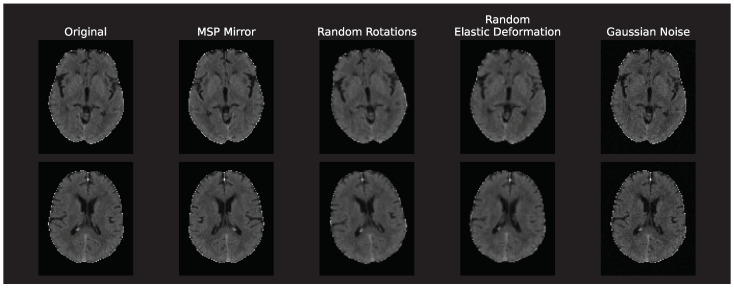
Axial slice examples of the augmentations used. The first and second rows show the axial slices 35 and 45, respectively, of the same 2 mm MNI152 template registered scan.

All Siamese network experiments were done with the DeepSymNet [24] as the base network. To recap, this Siamese network has two 3D encoders composed of four IMs, whose encodings are then merged by computing their L1 difference before being finally processed by two additional IMs and applying a global pooling operation that reduces the feature maps into feature vectors. We generalized this architecture by dividing it into three parts, as indicated by the architectural changes mentioned in the rightmost column of Figure 2:

1. Hemisphere encoder: A 3D CNN. The same encoders that were tried for the Baseline experiments were also tried here with the exception of the ResNet 50 [15].
2. Merger: Responsible for comparing the representations generated by the encoder. For example, the DeepSymNet [24] computes the L1 difference between the hemispheres representations, *before* applying the pooling operation and thus we call it Siamese-Before (row two of Figure 4). In contrast, in the Siamese-After approach, the L1 difference is computed after the global pooling is applied (row one of Figure 4).
  A third merge function, further explained in Figure A1 in the Appendix A, was considered. This approach tangles the features maps of the two encoders, and is named Siamese-Tangle, after this operation. Unlike the other two approaches, the Siamese-Tangle does not use the L1 Norm, but instead uses a learned comparison using group convolutions (due to memory limitations, the Siamese-Tangle experiment using the DeepSymNet encoder was run with a batch size of 16).
3. Global pooling: An operation which converts the feature maps (with spatial information) into feature vectors (without spatial information). We tried both global max pooling (GMP), which is the pooling operation used by the DeepSymNet [24], and global average pooling (GAP). Each operation convert the maps into features by computing the max and mean values of each map, respectively.

##### 3.3.1.3. Multiple Instance Learning (MIL)

The third and final architecture we tried for the image-only approach is based on the MIL paradigm. Unlike the previous two architectures that deal with 3D volumes, the MIL models have as input a bag of 2D axial slices (the instances), as can be seen in the third row of Figure 2. The axial plane was chosen over the other planes, because it is the most commonly used plane by physicians to inspect CT scans and it is also the plane used by the hemorrhage stroke detection MIL model proposed by Remedios et al. [29]. Following Ilse et al. [27], instances with many background pixels were removed. In the present work, slices with less than 100 pixels with a Hounsfield Unit (HU) value greater than 0 were removed, and therefore bags had a variable number of instances.

As depicted in the last column of Figure 2, we varied the encoders of the MIL models and also their aggregation function σ. Regarding the encoders, we tried a custom CNN and the ResNets 18, 34 and 50 [15]. The ResNets were used in two different settings: trained from scratch, but also used as feature extractors, leveraging their frozen ImageNet [40] pretrained weights from the timm open source library (Wightman, “PyTorch Image Models”, https://github.com/rwightman/pytorch-image-models accessed on 10 October 2022).

The aggregation functions σ tried were the instance vectors element wise mean (mean pooling), element wise max (max pooling) and MIL attention pooling, proposed by Ilse et al. [27].

#### 3.3.2. Hybrid Approach

Hybrid models take as input both tabular patient data and brain imaging data. There are many ways of combining these two data sources in a single model. For example, Ramos et al. [23] extracted features from CTs (using radiomics [41] and CNNs) and concatenated them with a set of tabular variables. Bacchi et al. [17] used a similar setup, but a feature extractor was also used on the tabular features. In both cases, the CT images were mapped into a feature vector, like we did in the image-only approach previously described.

**Figure 5:**
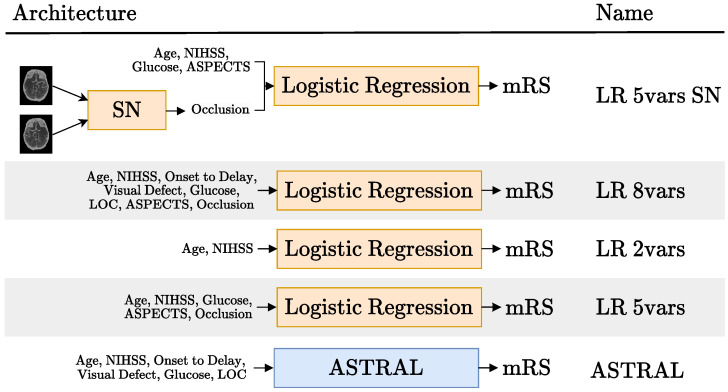
Schematic representation of the hybrid models tried (rows 1, 2 and 4) and the tabular models they were compared to (rows 3 and 5). The acronyms SN and LR stand for Siamese network and logistic regression, respectively. Each model is named after the number of variables it uses. The details regarding the SN block in the first row are available in Figure 8. Blocks in orange are updated during training and blocks in blue are not trained.

In our work, we try a different way of adding the imaging data to the tabular variables. Instead of using a CNN as a feature extractor, we use a CNN as an imaging biomarker classifier. In particular, we used a Siamese network to detect the presence or absence of occlusions in CTA scans, as shown in the first row of Figure 5. We call this model *LR 5vars SN*, because it is based on a logistic regression model (LR) with five variables, one of them being the occlusion, predicted with the Siamese network (SN). The four other variables are the patient age, baseline NIHSS, glucose levels and baseline ASPECTS score (also an imaging biomarker). In this work, we used expert annotations for this variable, but there are several published works [42,43] and commercial products [44,45] that predict the ASPECT score using machine learning.

The particular selection of five variables in the *LR 5vars SN* model came from a preliminary analysis of the feature importance of the *LR 8vars* experiment (second row of Figure 5). This LR model was trained on the six tabular variables used on the ASTRAL clinic classifier [10] (age, NIHSS, glucose, ASPECTS and occlusion, as can be seen in row 5 of Figure 5) and the two image biomarkers: ASPECTS and occlusion. The ASTRAL variables were chosen as a starting point because this classifier was originally adapted from a LR model [10] and also because, despite is simplicity, it has been proven hard to beat [3].

**Figure 6:**
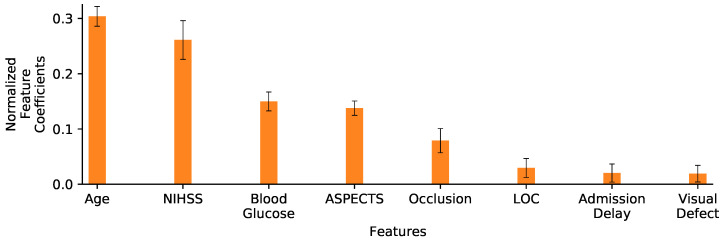
Normalized feature coefficients for the LR 8vars experiment. Values are displayed as the mean and standard deviation of coefficients learned on the 10 folds training sets. Variables are ordered by decreasing coefficient magnitude (importance).

Figure 6 displays the normalized feature coefficients (a proxy of feature importance) for the *LR 8vars* experiment. The relatively low importance LOC, admission delay and visual defect features is the reason why we did not include them in the *LR 5vars SN* experiment. This figure also shows how the age and the NIHSS variables are given notably more importance than the other variables, corresponding to more than half of the normalized feature coefficients by themselves. This prompted the *LR 2vars* experiment (Figure 5, row 3), where only these two variables are used.

We also included the *LR 5vars* experiment (Figure 5, row 4), which is exactly the same as the *LR 5vars SN* experiment, except for the fact that the occlusion biomarker comes from an expert annotation instead of the Siamese network. Finally, we also compared these results to the original ASTRAL classifier [10] (Figure 5, row 5).

Our original unprocessed tabular dataset had 743 rows (anonymized patients) and 511 columns. Of these columns, only the eight corresponding to the age, admission NIHSS score, onset-to-admission delay, visual field defect (NIHSS 3), blood glucose, decreased level of consciousness (LOC) (NIHSS 1A), ASPECTS and occlusion variables were used. Removing the patients with outlier and/or missing values reduced the dataset to just 413 individuals. This subset, comprising 270 and 143 individuals with good and poor outcome, respectively, was the one used for the experiments.

Cross validation with 10-folds was used, and the results are reported as the mean ± standard deviation of the metrics computed over the 10-folds test sets. In the training sets, the hyperparameters of the LRs were selected using Bayesian optimization [46]. The results of each experiment were measured with AUC and $F_{1}$-score and compared with paired *t*-tests, using a confidence level of α = 0.05, [47] to determine if the differences in their scores were statistically significant.

##### 3.3.2.1. Occlusion Prediction

As alluded to in the literature review, both Hilbert et al. [13] and Barman et al. [24] developed CNNs whose inputs are CTA scans for predicting the patients mRS and the presence of a stroke, respectively. Both authors note that their models end up being good occlusion detectors. Motivated by this observation, a model that predicts the occlusion variable was developed.

**Figure 7:**
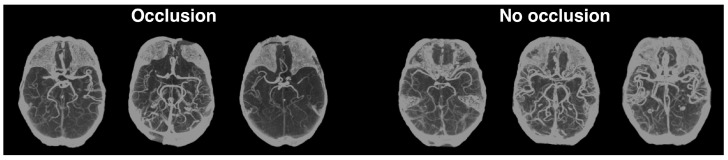
MIPs with (**left**) and without occlusions (**right**). This technique is here used to project the scans into a 2D image that highlights brain arteries in the axial plane.

Like Hilbert et al. [13], we also preprocessed our CTA scans using maximum intensity projection (MIP) for this particular task (for more details about the scans’ preprocessing, refer back to Section 3.2). We applied MIP between the axial slices 45 and 100 of the Montreal Neurological Institute (MNI) 1 mm template registered brains. This preprocessing results in a single 2D image for each patient, with this single view perpendicular to the axial plane. Examples of the application of this step can be seen in Figure 7. This figure also highlights how the hemisphere asymmetry in the brain arteries of the MIPs with occlusions is very noticeable, even for non-experts.

**Figure 8:**
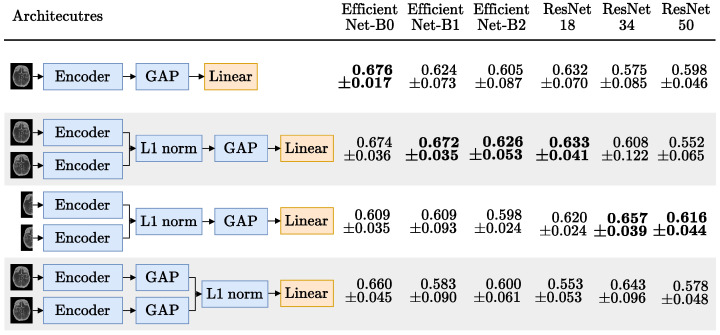
MIP occlusion mean ± standard deviation AUC, over the 5 test folds. Encoders, ordered by parameter count, have their respective highest AUC in bold. Blocks in blue are frozen or non trainable; only the linear layer with one output neuron, activated with a sigmoid, is updated during training.

This symmetry bias prompted the use of the Siamese approach, again. The different architectures tried (and their respective performance) are summarized in Figure 8. As the third row of this Figure shows, we tried feeding each hemisphere separately, like we did in the Siamese image-only models. We also tried feeding the whole image (flipped in one of the encoders), as shown in rows 2 and 4 of this figure. Finally, we also tried the baseline approach that uses a single encoder, as shown in the first row.

For each architecture in the rows of Figure 8 we used GMP and tried six different CNN encoders: the EfficientNets B0, B1 and B2 [39] and the ResNets 18, 34, 50 [15]. Hilbert et al. [13] used receptive field neural networks (RFNN) to avoid overfitting. To deal with this issue, and taking advantage of MIPs being 2D images, all the encoders tried had frozen weights, pretrained on ImageNet [40], using the implementation and weights provided by the timm open source library (Wightman, “PyTorch Image Models”, https://github.com/rwightman/pytorch-image-models, accessed on 10 October 2022).

Of the 361 patients with a CTA scan available (221 with occlusion and 140 without), 300 were used to train and 61 to test, using a stratified split. In the training set, 5 fold stratified cross validation was used to compare the architectures tried. All experiments were trained for 150 epochs, using the Adam optimizer [38], with binary cross entropy, a batch size of 32, weight decay of 0.001 and learning rate of 0.0002, multiplied by 0.1 every 50 epochs.

## 4. Results and Discussion

### 4.1. Image-Only Approach

#### 4.1.1. Baseline

**Figure 9:**
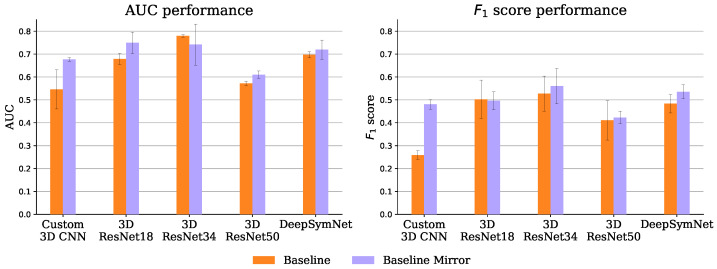
Baseline experiments mean and standard deviation AUC (**left**) and $F_{1}$-score (**right**), over the 3 test set runs.

Figure 9 shows the performance of the 10 experiments of the baseline approach. Despite being introduced somewhat naively, the brain hemisphere symmetry bias delivers interesting results. It improved both the AUC and the $F_{1}$-score for all encoders except the ResNet 18 and 34 (where only one of the metrics was improved and the other was closely matched). This bias is particularly useful for the lower capacity custom CNN, putting it on par with the ResNet18 without the bias, even though the ResNet has roughly 445 times more parameters.

Also on par with the ResNet 18 is the DeepSymNet encoder, in both the baseline and baseline mirror experiments, even though the ResNet has roughly 256 times more parameters. In fact, the 3D ResNets seem to have a relatively bad performance-parameters count trade-off, possibly due to their aggressive feature map subsampling.

#### 4.1.2. Siamese Network

Figure 4 shows the AUC scores obtained by each image-only Siamese experiment, when using GMP. It was expected that the Siamese-After approach would underperform relatively to the other approaches, because they compare the encodings at the feature map level. The spatial information they leverage should be important, since the ischemic signs location are suggestive of the occlusion site, which is a strong functional outcome predictor [9]. As shown in this Figure, using GMP, indeed the Siamese-Tangle approach performed better than the other two for all the encoders, but the 3D ResNet 18. However, the expectations were not met for the Siamese-Before method that was outperformed by the Siamese-After method for all encoders but the 3D ResNet 34 (where the two approaches are closely matched).

**Figure 10:**
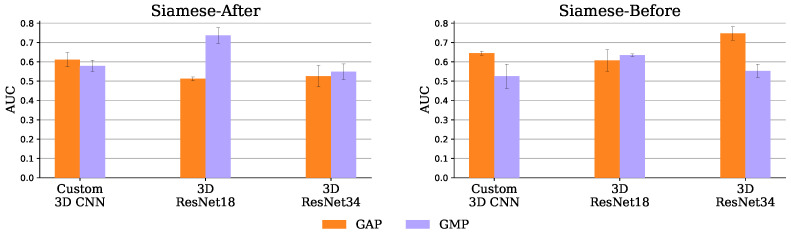
Siamese-After (**left**) and Siamese-Before (**right**) mean and standard deviation AUC, over the 3 test set runs, with GAP and GMP.

This may not be an issue of the Siamese-Before method itself, but rather of its synergy with the GMP. As can be seen in Figure 10, which compares the two pooling operators tried (GMP and GAP), the GAP seems to suit the Siamese-Before better. With GAP, the Siamese-Before does indeed outperform the Siamese-After method for the three encoders tested. This may happen because with GAP every value in the feature map is considered, whereas with GMP, only the maximum value per map is considered.

The Siamese-Before with a 3D ResNet 34 encoder and GAP had the best AUC of 0.747 ± 0.035. Both baseline and Siamese runs obtained over 95% accuracy in the training set, a performance that did not generalize to the test set. This overfitting may have affected the Siamese models even more given their increased capacity due to the extra hemisphere comparison encoder. Nevertheless, this potential excessive capacity was not arbitrary, as the architectures tried were based on the successful DeepSymNet [24] trained on a dataset of comparable size and similar training hyper parameters.

#### 4.1.3. Multiple Instance Learning (MIL)

**Figure 11:**
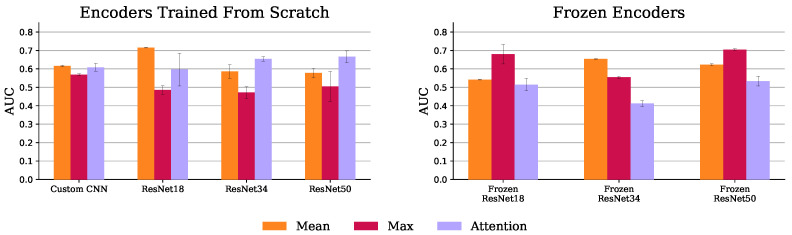
MIL mean and standard deviation AUC, over the 3 test set runs, with trained (**left**) and frozen (**right**) encoders.

**Figure 12:**
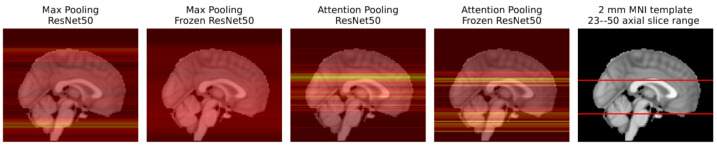
Sagittal view of the axial slice importance for the ResNet 50 experiments, using max and attention MIL pooling, in the test set examples. Brighter colors represent more important slices. For the max experiments, the importance of a slice was considered to be the average count of max features that slice contributed to the aggregated vector. For the attention experiments, the importance of each slice is simply the average value given by the attention pooling to that slice. The heatmaps are superimposed on the 2 mm MNI152 [30] template, also plotted on the right with the middle cerebral artery (MCA) range highlighted in red.

Figure 11 shows the AUC scores for all MIL experiments. The MIL attention pooling is a generalization of the mean pooling (attention pooling can be seen as a weighted mean), thus it should be able to perform at least as well as the mean pooling. Indeed, as shown in this figure, for all the trained from scratch experiments, the attention pooling was on par or better than the mean pooling, except when using the ResNet 18. However, with pretrained encoders, its performance drastically decreases. This may not be a problem of the pretrained features, but rather of the instances the attention pooling is focusing on using these encoders. As Figure 12 shows, with the frozen ResNet 50, the attention pooling focuses on several slices at the level of the cerebellum. Although this region can have relevant information about the target variable, physicians tend to concentrate on the slices in the middle cerebral artery (MCA) range (the area inspected by the ASPECT score [8], for example). The edge of the attention pooling over the mean pooling is only materialized when the network is able to focus on these more relevant regions.

The max pooling models with encoders trained from scratch had the worst results. As Figure 12 shows, this experiment, using a ResNet 50, lays emphasis at the less informative MCA range complement. Conversely, when these models have their encoders frozen, their performance improves as the slice importance is spread over the whole volume. Indeed, equally spreading the attention over all instances, like the mean pooling aggregator does, provides a decent baseline solution.

### 4.2. Hybrid Approach

The AUC and $F_{1}$-score performance obtained for the experiments depicted in Figure 5 are presented in Table 2. As can be seen in this table, the *LR vars SN* did outperform the ASTRAL classifier both in terms of AUC and $F_{1}$-score. However, this performance difference is marginal and, indeed, not statistically significant, as can be seen in Table 3 and Table 4. These tables display the *p*-values of the *t*-tests: 0.327 and 0.986 for the comparison in AUC and $F_{1}$-score, respectively, of these first two models.

Table 3 has the *p*-values of the paired *t*-tests regarding the AUC scores. This table shows that the performance difference between the *LR 5vars* experiment and the ASTRAL classifier was statistically significant. On the other hand, even though the *LR 5vars SN* experiment was also able to outperform the ASTRAL classifier, this difference was not significant. Table 4 has the same information shown in Table 3, but for the $F_{1}$-score evaluation. Using this metric, no score was able to significantly outperform the ASTRAL classifier. Finally, what is perhaps the most interesting result is that no experiment had a performance statistically different from the very simple *LR 2vars* experiment, both in terms of AUC and $F_{1}$-score. It may seem strange that the AUC score difference between the *LR 5vars* and *LR 8vars* experiments is statistically significant when it is not between the *LR 5vars* and *LR 2vars* experiments (given that, just looking at Table 2, the latter performance difference is larger). Looking at Table 3 and Table 4, there are other situations like this one. However, it is important to keep in mind that the paired *t*-test checks for differences on the individual fold performance differences, not the overall performance difference.

#### Occlusion Prediction

Figure 8 has the results for the occlusion prediction experiments. Here, the Siamese bias seems to have worked as expected since, for all encoders, either the first or the second Siamese approach outperformed the baseline, except for the EfficientNet-B0 (which is closely matched by the first Siamese approach). This is also the encoder with the best AUC, despite being the smallest (even outperforming the larger EfficientNets B1 and B2).

The Siamese with the EfficientNet-B0 encoder outperformed the EfficientNets B1 and B2 possibly because the MIP images are 218 × 182 and these models were pretrained with larger images. This also explains why the performance using these encoders is worse on the second Siamese version, where the image input size is even smaller (as the MIPs are cut in half). Still, at least for the first approach, where the MIP size is close to the usual 224 × 224 ImageNet [40] input size, the EfficientNets outperformed the ResNets which is consistent with the finding that higher ImageNet accuracy translates into higher transfer learning accuracy [48]. Also, again, the Siamese-Before approach (second and third rows) outperformed the Siamese-After approach (last row), further showing the importance of the spatial dimension of the feature maps in the hemisphere comparison.

Finally, the best-performing Siamese model (first version with an EfficientNet-B0 encoder) was selected and trained with the whole training set. This trained model was then evaluated on the 61 scans reserved for the test set, obtaining a 0.753 AUC and 0.790 $F_{1}$-score. This is the model that was used for the hybrid *LR 5vars SN* experiment.

## 5. Discussion

### 5.1. Image-Only Approach Underperformance

In a traditional image classification problem, the classification setting is usually well defined. For example, in ImageNet, an image has a class “cat” if the main object in the image is indeed a cat. The same is true for the hemorrhagic stroke detection algorithm proposed by Remedios et al. [29] or the ischemic stroke detection CNN DeepSymNet [24]: a CT scan has a positive label if it contains visible stroke signs and has a negative label otherwise. On the other hand, when using the image-only approach for the prediction of the mRS variable, this classification setting is less clear. In this setting, a CT scan has a positive label if it belongs to a patient that will have a poor outcome, three months in the future. In the case of stroke detection, one can point to specific regions of the brain that look damaged and objectively argue that they belong to a stroke patient. In the case of mRS prediction, even when such brain damage signs are visible, there are no guarantees that they will be materialized into a bad outcome, three months later (i.e., the label is not objectively determined solely from the image).

The vagueness of this problem definition not only makes it more difficult to optimize, but also produces solutions that are less interpretable. Indeed, there are visualization methods (e.g., GradCAM [49]) that make it possible to inspect where the neural network is “looking at” to make its prediction. These methods can be useful to detect if the network is not “cheating” by finding some sporadic or accidental correlation with the target variable. However, besides that, just knowing the brain region the network is focusing on does not explain why that region would entail a good or poor outcome.

Another reason for the relative underperformance of the image-only approach is that these models do not have access to important data used by the tabular and hybrid models, like the age and NIHSS variables. According to our feature importance analysis, as well as several other authors [3,10,11,12,13,23], the age and NIHSS are strong mRS predictors. On the one hand, even without a stroke, the age is already a good predictor of someone’s independence as people tend to lose their autonomy as they grow older. Additionally, older people are less likely to recover from a stroke [50]. On the other hand, the NIHSS directly measures the suffered neurological deficits, even evaluating the patient’s leg and arm motor abilities, which are essential to perform daily tasks. In other words, what is measured by the NIHSS is very closely related to what is measured by the mRS. Finally, as the stroke symptoms are maximal on onset and become less severe over time, high NIHSS scores registered several hours/days after the stroke are even more correlated with the Rankin scale [51].

### 5.2. Imaging Biomarkers

The prediction of image biomarkers does not suffer from the fuzzy definition that affects the image-only mRS prediction approach. For example, the ASPECTS is scored according to the status of ten different MCA regions, which are graded by looking at NCCT scans. Another example is the presence of absence of occlusions: either there is a vessel occluded or not. This leads to an easier optimization process, as previously argued, and as witnessed by the better results we obtained in the occlusion prediction task compared to the mRS prediction task results.

The problem with predicting biomarkers is that our original goal was to predict the mRS score, not some other biomarker. That said, these biomarkers can be used that improve the prediction of our original target variable, as we have shown in our hybrid experiments. Another advantage of predicting an imaging biomarker as an intermediate step to incorporate imaging data to an hybrid model is increased interpretability (an important factor for the deployment in a real medical setting). Bacchi et al. [17], Samak et al. [18] and Ramos et al. [23] incorporated imaging data in their hybrid models using CNNs. In all of these works, this was done by extracting a set of visual features, which comprise a vector of (a few hundred) numbers that are concatenated with the other tabular features and fed into a classifier that outputs the predicted mRS. The problem with this approach is that these visual features are not human-readable. In other words, there is rarely a clear link between each number in the visual feature vector and a corresponding visual characteristic in the original CT (this is the reason why deep learning models are viewed as black boxes). The intermediate prediction of a biomarker alleviates this problem by outputting a prediction that has, by definition, a visual interpretation.

### 5.3. Feature Selection

The best performing experiment was the *LR 5vars*, both in terms of AUC and $F_{1}$-score. The AUC is the most widely used metric to assess the performance of these prognostic models, despite being known that it provides unreliable estimates in low sample size and class imbalanced regimes [52] (characteristics of the present dataset and of those previously used in other studies). For this reason, the AUC results should be used with caution, and that is why they are here accompanied with the $F_{1}$-score. Indeed, using this metric, the *LR 5vars* experiment was not significantly better than the ASTRAL classifier, even though it was four percentage points higher, on average.

Compared with previous works that also used ML [3,53,54], the *LR 5vars* experiment uses very few input features, as these models use dozens of variables. This may help explain its performance, as feature selection is known to be an important step for ML models [55]. Additionally, having few variables is again something important for the actual applicability of these models in practice.

If using few variables is a good thing, it may be tempting to simply use the *LR 2vars* model, specially as no other model had significant performance difference compared to it. However, it is worth noting that it had the lowest $F_{1}$-score and that its performance difference regarding this metric was close to be significant for the ASTRAL and *LR 5vars* experiments. Still, this score is competitive with or better than various previously published algorithms that use the tabular [3,53], image-only [13,21] and hybrid [17,18] approaches.

### 5.4. Limitations

One of the limitations of our study is the small size of the datasets used to train our models. The image-only models were trained on only 365 images, which is a relatively small dataset. While we employed data augmentation, the model training may have been impacted by the limited number of raw samples. In particular, Remedios et al. [29] note how at least 400 samples were needed to achieve model generalization in their hemorrhage detection task. Additionally, detecting hemorrhage in NCCTs is arguably a much easier task than mRS prediction, not only because this task is better defined, but also because NCCTs are regarded as highly sensitive to the presence of hemorrhage, but not as sensitive to acute ischemic signs [7]. In the occlusion prediction network, we only had 300 CTA images available, but this lack of data were somewhat compensated by the use of transfer learning, enabled by the use of MIP to project the scans into 2D images.

The MIP technique has some drawbacks too. Depth perception and information about arteries perpendicular to the axial plane (like the anterior cerebral artery) and outside the range considered in MIP (like the basilar artery) may be lost using this projection, leading to classification errors. Additionally, stenoses may also disturb the brain symmetry the Siamese models rely on to make predictions, eventually leading to misclassifications. One possible way of addressing these limitations is to include other MIP views perpendicular to other anatomical planes, essentially representing each patient by various MIP images, instead of a single one.

Another limitation is the fact that we used expert labeled ASPECT scores [8] in the hybrid experiments. Ideally, such labels would have been predicted using a deep learning model to better illustrate the point that deep learning can be used to extract biomarkers to improve the prediction of the functional outcome of ischemic stroke patients. Unfortunately we did not have enough labeled samples to build our own ASPECTs model, and we did not use any of the ASPECTs prediction commercial products [44,45].

Finally, analyzing the multicollinearity of the variables of the hybrid experiments, using variance inflation factor (VIF) reveals that experiments *LR 5vars*, *LR 5vars SN* and *LR 8vars* have some variables with a VIF greater than 10. This does not affect the overall performance of these models but may affect their individual predictions which is very important if they are going to be deployed in a real setting.

### 5.5. Future Work

Although this work shows how the biomarkers predicted from CT images can help on the mRS prognostic task, the prediction of these biomarkers still needs improvement. Notably, the performance difference between the *LR 5var SN* and the ASTRAL experiments was not statistically significant. Training a model to predict several of these biomarkers at once in a multi-task setting can not only be helpful in low sample regimes [56], but also computationally cheaper.

## 6. Conclusions

In this study, for the imaging-only approach, we presented three different architectures, which include the Siamese network and MIL architectures, novel in the context of mRS prediction. We also presented some hybrid models whose inputs are clinical and demographic variables, as well as two imaging biomarkers: ASPECTS and occlusion (the latter being automatically predict from CTA scans with a CNN). The hybrid models obtained a better performance than the image-only models, which is in accordance to the results previously reported in the literature. Finally, we discussed limitations of directly predicting outcomes from images alone, which may explain the inferior performance of this approach. We proposed an intermediate step of extracting imaging biomarkers using deep learning first, then incorporated these quantitative image features into prognostic models. This approach could help address challenges faced by end-to-end image classification techniques for outcome prediction, and also make the resulting model more interpretable.

## Figures and Tables

**Table 1:** Demographic, clinical and imaging statistics of the cohort. The acronym LOC means level of consciousness, and was obtained from the parameter 1A of the NIHSS score.

	Occurrence (%) *N* = 743	Missing *n* (%)
ASTRAL [10] Variables		
Age in years, median (IQR)	71 (57–80)	11 (1)
NIHSS, median (IQR)	7.5 (3–14)	45 (6)
Onset-to-admission delay in hours, median (IQR)	2 (1–5)	205 (28)
Visual Defect, *n* (%)	286 (38)	45 (6)
LOC, *n* (%)	59 (8)	45 (6)
Blood Glucose in milligrams per deciliter, median (IQR)	120 (101–154)	142 (19)
Imaging Variables		
ASPECTS, median (IQR)	10 (8–10)	156 (21)
Occlusion, *n* (%)	377 (50)	48 (6)
Other Variables (not considered in the analysis)		
Female sex, *n* (%)	307 (41)	6 (1)
Arterial Hypertension, *n* (%)	531 (71)	20 (3)
Diabetes, *n* (%)	553 (74)	16 (2)
Previous Ischemic Stroke, *n* (%)	112 (15)	19 (3)
Ischemic Heart Disease, *n* (%)	97 (13)	40 (5)
Outcome Variable		
90-day mRS, median (IQR)	1 (0–3)	0 (0)
90-day mRS > 2, *n* (%)	252 (34)	0 (0)

**Table 2:** Features and performance, in AUC and $F_{1}$-score, of the different hybrid and tabular experiments, described in Figure 5.

Model Name	AUC	$F_{1}$-Score
LR 5vars SN	0.806± 0.082	0.611 ± 0.113
LR 8vars	0.796 ± 0.077	0.602 ± 0.118
LR 2vars	0.791 ± 0.090	0.586 ± 0.135
LR 5vars	0.809 ± 0.084	0.646 ± 0.141
ASTRAL	0.784 ± 0.099	0.601 ± 0.115

**Table 3:** AUC paired *t*-test *p*-values for the 5 experiments. The table is only half filled, as the other half is symmetric. Cells where the *p*-value is less than α (0.05) are marked with *. The * marks cells where the row experiment has a lower score than the column experiment.

	LR 8vars	LR 2vars	LR 5vars	LR 5vars SN
ASTRAL	0.327	0.471	0.024 *	0.062
LR 8vars		0.681	0.021 *	0.039 *
LR 2vars			0.130	0.216
LR 5vars				0.128

**Table 4:** $F_{1}$-score paired *t*-test *p*-values for the 5 experiments. The table is only half filled, as the other half is symmetric. Cells where the *p*-value is less than α (0.05) are marked with * or ^†^. The */^†^ mark cells where the row experiment has a lower/great score than the column experiment, respectively.

	LR 8vars	LR 2vars	LR 5vars	LR 5vars SN
ASTRAL	0.986	0.088	0.191	0.743
LR 8vars		0.642	0.028 *	0.505
LR 2vars			0.087	0.415
LR 5vars				0.021 ^†^

## Data Availability

The data used in this study came from the PRECISE study (“PRECISEMED”, https://www.precisemed.org/), which is not publicly available. However, we make available the code used in this study. A link to our GitHub page is available in Appendix C.

## Abbreviations

    The following abbreviations are used in this manuscript:

ANN	artificial neural network
ASPECT	Alberta stroke programme early computed tomography
ASTRAL	Acute Stroke Registry and Analysis of Lausanne
AUC	area under the curve
BET	brain extraction tool
CNN	convolutional neural network
CT	computed tomography
CTA	computed tomography angiography
DICOM	Digital Imaging and Communications in Medicine
FLIRT	FMRIB’s Linear Image Registration Tool
FSL	FMRIB Software Library
GAP	global average pooling
GMP	global max pooling
HU	Hounsfield unit
IM	inception module
INR	international normalized ratio
LOC	level of consciousness
LR	logistic regression
MCA	middle cerebral artery
MIL	multiple instance learning
MIP	maximum intensity projection
ML	machine learning
MNI	Montreal Neurosciences Institute
MSP	mid-sagittal plane
NCCT	non-contrast computed tomography
NIHSS	National Institutes of Health Stroke Scale
NIfTI	Neuroimaging Informatics Technology Initiative
RFNN	receptive field neural network
ROC	receiver operating characteristic
ReLU	rectified linear unit
mRS	Modified Rankin Scale
timm	Pytorch Image Models

## Appendix A. Merge Function

Figure A1 elaborates the tangle operation that was referenced in the Siamese network, image-only experiments, described in Section 3.3.1.2.

## Appendix B. NCCT Augmentations

Figure A2 has the results of applying the NCCT augmentations, mentioned in Section 3.2, to a given NCCT sample. It may seem counterintuitive to apply random rotations after the template registration step where all scans are put in the same position. However, the registration is not always perfect and brains are not equally well aligned after this step. Random rotations are therefore included to help make the models robust to these imperfections.

## Appendix C. Implementation Details

The code for all experiments can be found here https://github.com/GravO8/mrs-dl, accessed on 12 October 2023.

All neural networks were implemented using the PyTorch library [58], version 1.10.2+cu102.

The statistical tests were performed using the ttest_rel function from the SciPy library [59], version 1.4.1.

## Appendix D. Image-Only Experiments Results Tables

**Table A1:** Image-only experiments accuracy and AUC scores over the 3 test sets, round to three decimal places.

Experiment Name	Parameters	Accuracy	AUC
Baseline	Name = “Baseline”, Encode = “3D Custom CNN”	0.622 ± 0.059	0.546 ± 0.085
Baseline	Name = “Baseline”, Encode = “3D ResNet 18”	0.728 ± 0.051	0.679 ± 0.024
Baseline	Name = “Baseline”, Encode = “3D ResNet 34”	0.711 ± 0.035	0.779 ± 0.006
Baseline	Name = “Baseline”, Encode = “3D ResNet 50”	0.528 ± 0.042	0.572 ± 0.010
Baseline	Name = “Baseline”, Encode = “DeepSymNet Encoder”	0.694 ± 0.010	0.697 ± 0.014
Baseline	Name = “Baseline Mirror”, Encode = “3D Custom CNN”	0.567 ± 0.067	0.677 ± 0.008
Baseline	Name = “Baseline Mirror”, Encode = “3D ResNet 18”	0.689 ± 0.025	0.749 ± 0.047
Baseline	Name = “Baseline Mirror”, Encode = “3D ResNet 34”	0.767 ± 0.033	0.741 ± 0.090
Baseline	Name = “Baseline Mirror”, Encode = “3D ResNet 50”	0.617 ± 0.073	0.610 ± 0.016
Baseline	Name = “Baseline Mirror”, Encode = “DeepSymNet Encoder”	0.656 ± 0.051	0.719 ± 0.042
Siamese Network	Name = “Siamese After”, Encoder = “3D Custom CNN”, Global Pooling = “GMP”	0.617 ± 0.050	0.579 ± 0.030
Siamese Network	Name = “Siamese After”, Encoder = “3D ResNet 18”, Global Pooling = “GMP”	0.600 ± 0.060	0.736 ± 0.041
Siamese Network	Name = “Siamese After”, Encoder = “3D ResNet 34”, Global Pooling = “GMP”	0.483 ± 0.173	0.549 ± 0.040
Siamese Network	Name = “Siamese After”, Encoder = “DeepSymNet Encoder”, Global Pooling = “GMP”	0.572 ± 0.035	0.609 ± 0.062
Siamese Network	Name = “Siamese After”, Encoder = “3D Custom CNN”, Global Pooling = “GAP”	0.644 ± 0.042	0.611 ± 0.037
Siamese Network	Name = “Siamese After”, Encoder = “3D ResNet 18”, Global Pooling = “GAP”	0.567 ± 0.000	0.513 ± 0.010
Siamese Network	Name = “Siamese After”, Encoder = “3D ResNet 34”, Global Pooling = “GAP”	0.511 ± 0.146	0.526 ± 0.055
Siamese Network	Name = “Siamese After”, Encoder = “DeepSymNet Encoder”, Global Pooling = “GAP”	0.661 ± 0.048	0.668 ± 0.079
Siamese Network	Name = “Siamese Before”, Encoder = “3D Custom CNN”, Global Pooling = “GMP”	0.600 ± 0.017	0.525 ± 0.063
Siamese Network	Name = “Siamese Before”, Encoder = “3D ResNet 18”, Global Pooling = “GMP”	0.650 ± 0.073	0.634 ± 0.007
Siamese Network	Name = “Siamese Before”, Encoder = “3D ResNet 34”, Global Pooling = “GMP”	0.622 ± 0.054	0.553 ± 0.034
Siamese Network	Name = “Siamese Before”, Encoder = “DeepSymNet Encoder”, Global Pooling = “GMP”	0.600 ± 0.017	0.556 ± 0.021
Siamese Network	Name = “Siamese Before”, Encoder = “3D Custom CNN”, Global Pooling = “GAP”	0.583 ± 0.117	0.645 ± 0.010
Siamese Network	Name = “Siamese Before”, Encoder = “3D ResNet 18”, Global Pooling = “GAP”	0.661 ± 0.084	0.607 ± 0.056
Siamese Network	Name = “Siamese Before”, Encoder = “3D ResNet 34”, Global Pooling = “GAP”	0.744 ± 0.069	0.747 ± 0.035
Siamese Network	Name = “Siamese Tangle”, Encoder = “3D Custom CNN”, Global Pooling = “GMP”	0.611 ± 0.086	0.665 ± 0.068
Siamese Network	Name = “Siamese Tangle”, Encoder = “3D ResNet 18”, Global Pooling = “GMP”	0.561 ± 0.077	0.653 ± 0.043
Siamese Network	Name = “Siamese Tangle”, Encoder = “3D ResNet 34”, Global Pooling = “GMP”	0.600 ± 0.076	0.682 ± 0.057
Siamese Network	Name = “Siamese Tangle”, Encoder = “DeepSymNet Encoder”, Global Pooling = “GMP”	0.706 ± 0.042	0.689 ± 0.038
MIL	Encoder = “2D Custom CNN”, Frozen ImageNet Weights = “N/A”, σ = “Max”	0.583 ± 0.017	0.569 ± 0.006
MIL	Encoder = “ResNet 18”, Frozen ImageNet Weights = “No”, σ = “Max”	0.561 ± 0.059	0.486 ± 0.023
MIL	Encoder = “ResNet 18”, Frozen ImageNet Weights = “Yes”, σ = “Max”	0.583 ± 0.044	0.680 ± 0.053
MIL	Encoder = “ResNet 34”, Frozen ImageNet Weights = “No”, σ = “Max”	0.472 ± 0.063	0.472 ± 0.032
MIL	Encoder = “ResNet 34”, Frozen ImageNet Weights = “Yes”, σ = “Max”	0.450 ± 0.029	0.554 ± 0.005
MIL	Encoder = “ResNet 50”, Frozen ImageNet Weights = “No”, σ = “Max”	0.461 ± 0.059	0.505 ± 0.081
MIL	Encoder = “ResNet 50”, Frozen ImageNet Weights = “Yes”, σ = “Max”	0.706 ± 0.010	0.705 ± 0.005
MIL	Encoder = “2D Custom CNN”, Frozen ImageNet Weights = “N/A”, σ = “Mean”	0.289 ± 0.025	0.616 ± 0.003
MIL	Encoder = “ResNet 18”, Frozen ImageNet Weights = “No”, σ = “Mean”	0.672 ± 0.067	0.715 ± 0.001
MIL	Encoder = “ResNet 18”, Frozen ImageNet Weights = “Yes”, σ = “Mean”	0.294 ± 0.010	0.542 ± 0.001
MIL	Encoder = “ResNet 34”, Frozen ImageNet Weights = “No”, σ = “Mean”	0.656 ± 0.025	0.586 ± 0.036
MIL	Encoder = “ResNet 34”, Frozen ImageNet Weights = “Yes”, σ = “Mean”	0.617 ± 0.000	0.653 ± 0.002
MIL	Encoder = “ResNet 50”, Frozen ImageNet Weights = “No”, σ = “Mean”	0.528 ± 0.054	0.578 ± 0.025
MIL	Encoder = “ResNet 50”, Frozen ImageNet Weights = “Yes”, σ = “Max”	0.711 ± 0.010	0.623 ± 0.004
MIL	Encoder = “2D Custom CNN”, Frozen ImageNet Weights = “N/A”, σ = “Attention”	0.633 ± 0.000	0.608 ± 0.021
MIL	Encoder = “ResNet 18”, Frozen ImageNet Weights = “No”, σ = “Attention”	0.589 ± 0.042	0.597 ± 0.089
MIL	Encoder = “ResNet 18”, Frozen ImageNet Weights = “Yes”, σ = “Attention”	0.422 ± 0.010	0.515 ± 0.034
MIL	Encoder = “ResNet 34”, Frozen ImageNet Weights = “No”, σ = “Attention”	0.706 ± 0.025	0.654 ± 0.013
MIL	Encoder = “ResNet 34”, Frozen ImageNet Weights = “Yes”, σ = “Attention”	0.378 ± 0.019	0.412 ± 0.016
MIL	Encoder = “ResNet 50”, Frozen ImageNet Weights = “No”, σ = “Attention”	0.650 ± 0.033	0.666 ± 0.032
MIL	Encoder = “ResNet 50”, Frozen ImageNet Weights = “Yes”, σ = “Attention”	0.611 ± 0.042	0.534 ± 0.027
